# Age-Related Gene Alteration in Naïve and Memory T cells Using Precise Age-Tracking Model

**DOI:** 10.3389/fcell.2020.624380

**Published:** 2021-02-11

**Authors:** Xiaofeng Yang, Xin Wang, Lei Lei, Lina Sun, Anjun Jiao, Kun Zhu, Tao Xie, Haiyan Liu, Xingzhe Zhang, Yanhong Su, Cangang Zhang, Lin Shi, Dan Zhang, Huiqiang Zheng, Jiahui Zhang, Xiaobin Liu, Xin Wang, Xiaobo Zhou, Chenming Sun, Baojun Zhang

**Affiliations:** ^1^Department of Pathogenic Microbiology and Immunology, School of Basic Medical Sciences, Xi'an Jiaotong University, Xi'an, China; ^2^Institute of Infection and Immunity, Translational Medicine Institute, Xi'an Jiaotong University Health Science Center, Xi'an, China; ^3^Key Laboratory of Environment and Genes Related to Diseases (Xi'an Jiaotong University), Ministry of Education, Xi'an, China; ^4^Department of Biochemistry and Molecular Biology, University of Georgia, Athens, GA, United States; ^5^Center for Molecular Medicine, University of Georgia, Athens, GA, United States

**Keywords:** aged T cells, naïve T cells, *TCR*δ^*CreER*^*R26*^*ZsGreen*^ mice, CD4 T cells, CD8 T cells

## Abstract

In aged individuals, age-related changes in immune cells, especially T cell deficiency, are associated with an increased incidence of infection, tumor, and autoimmune disease, as well as an impaired response to vaccination. However, the features of gene expression levels in aged T cells are still unknown. Our previous study successfully tracked aged T cells generated from one wave of developing thymocytes of young age by a lineage-specific and inducible Cre-controlled reporter (*TCR*δ^*CreER*^*R26*^*ZsGreen*^ mouse strain). In this study, we utilized this model and genome-wide transcriptomic analysis to examine changes in gene expression in aged naïve and memory T cell populations during the aging process. We identified profound gene alterations in aged CD4 and CD8 T cells. Both aged CD4^+^ and CD8^+^ naïve T cells showed significantly decreased organelle function. Importantly, genes associated with lymphocyte activation and function demonstrated a significant increase in aged memory T cells, accompanied by upregulation of immunosuppressive markers and immune checkpoints, revealing an abnormal T cell function in aged cells. Furthermore, aging significantly affects T cell survival and death signaling. While aged CD4 memory T cells exhibited pro-apoptotic gene signatures, aged CD8 memory T cells expressed anti-apoptotic genes. Thus, the transcriptional analysis of gene expression and signaling pathways in aged T cell subsets shed light on our understanding of altered immune function with aging, which will have great potential for clinical interventions for older adults.

## Introduction

Age-associated progressive loss of physiological integrity may lead to major human pathologies, including cancer, cardiovascular disorders, diabetes, and neurodegenerative diseases. Age-related declines in the immune system, known as immunosenescence, lead older individuals to be more susceptible to infectious diseases, tumors, and autoimmune diseases, while their response to vaccination is impaired. As a key component of the immune system, T cell immunity during the aging process has attracted much attention in recent years (Nikolich-Zugich, 2018).

Naïve T cells develop in the thymus gland, which experiences rapid involution after puberty. To compensate for the reduced thymic export, naïve T cells maintain their population through peripheral homeostatic proliferation in the elderly. Although homeostatic proliferation is sufficient to maintain a sizable naïve CD4^+^ T cell compartment, loss of circulating naïve CD8^+^ T cells with age is much more severe (Thome et al., 2016). The exact mechanism underlying the reduced naïve CD8^+^ T cell compartment due to aging remains poorly understood. Once exposed to antigen, naïve T cells become activated and differentiate into effector and memory T cells. Based on their distinct homing capacity and effector function, memory T cells are further divided into central memory T (TCM) cells and effector memory T (TEM) cells. Along with aging, T cell subset distribution shifts from naïve T cells to TCM and TEM due to continuous antigen stimulation and thymic involution (Saule et al., 2006).

The aging process is accompanied by immunosenescence, which is associated with the loss of expression of co-stimulatory molecules, such as CD27 and CD28, and the reduction in IL-2 secretion (Li et al., 2019). It has been shown that exposure to short-term and long-term stress can induce T cell senescence, and cellular senescence is generally implicated as a major mechanism of aging-associated T cell dysfunction (Vermes et al., 1995). Immunosenescence reduces recognition of new antigens due to decreased TCR variability, which contributes to increased susceptibility to infection and ineffective response to vaccination in aged individuals (Dorrington and Bowdish, 2013). T cell senescence is also associated with increased pro-inflammatory cytokine production, which is known as inflammaging. In addition, DNA damage, such as double-strand breaks, inefficient repair, and reduced telomerase activity, are also enriched in aged T cells. The responses resulting from chronic DNA damage may contribute to the production of pro-inflammatory cytokines (Krysko et al., 2008).

Studies on the relationship between changes in gene expression and T cell function are essential for a better understanding of age-associated T cell immunity. In the current investigation, we have creatively applied *TCR*δ^*CreER*^*R26*^*ZsGreen*^ double transgenic mice for precise age-tracking and T cell sorting (Zhang et al., 2016). RNA sequencing (RNA-Seq) was performed among young and aged T cell populations, including both CD4 and CD8 T lymphocytes, and naïve and memory cell subsets in young and old mice. We analyzed differential gene expression patterns in the aged T cell population and identified a large number of genes involved in cellular and molecular functions, protein activity, nucleotide binding, and cell adhesion during the aging process. Notably, aged memory T cells exhibited gene patterns of abnormal immune functions. Aged CD4 and CD8 memory T cells showed gene signatures that were prone to cell death and resistant to cell death, respectively.

## Materials and Methods

### Mice

Five-week-old *TCR*δ^*CreER*^*R26*^*ZsGreen*^ mice were developed and used as described previously (Zhang et al., 2016). All mice were housed under specific pathogen-free conditions by the Xi'an Jiaotong University Division of Laboratory Animal Research. All animal procedures were approved by the Institutional Animal Care and Use Committee of Xi'an Jiaotong University.

### Antibodies

The following antibodies were used in the flow cytometry analyses: anti-mouse CD8a (53-6.7), anti-mouse CD4 (GK1.5), anti-mouse CD44 (IM7), anti-mouse 62L (MEL-14), anti-mouse CD69 (H1.2F3), anti-mouse CD122 (TM-βTM), and anti-mouse PD-1 (29F.1A12). The fluorochrome-labeled antibodies were purchased from BioLegend (San Diego, CA, USA).

#### Tamoxifen Treatment

For long-term tracing experiments, *TCR*δ^*CreER*^*R26*^*ZsGreen*^ mice (5 weeks old, *n* = 10) were treated with three doses of 1 mg/mouse tamoxifen intraperitoneal injection (i.p.) every other day. For short-term tracing experiments, *TCR*δ^*CreER*^*R26*^*ZsGreen*^ mice (5 weeks old, *n* = 8) were treated with one dose of tamoxifen (1 mg/mouse).

#### Flow Cytometry Analysis and Sorting

Lymphocytes from tamoxifen-treated *TCR*δ^*CreER*^*R26*^*ZsGreen*^ double transgenic mice were isolated. For cell surface staining, single cells were suspended, and a total of 1 × 10^6^ cells were stained in the dark at 4°C for 30 min. The analysis was performed on a CytoFLEX flow cytometer (Beckman Coulter, Brea, CA, USA). ZsGreen^+^ CD4 TEM (CD4^+^CD44^+^CD62L^−^), CD4 TCM (CD4^+^CD44^+^CD62L^+^), CD4 naïve T cells (CD4^+^CD44^−^CD62L^+^CD69^−^CD122^−^PD-1^−^), and CD8 TEM (CD8^+^CD44^+^CD62L^−^), CD8 TCM (CD4^+^CD44^+^CD62L^+^), and CD8 naïve T cells (CD8^+^CD44^−^CD62L^+^CD69^−^CD122^−^PD-1^−^) were sorted by MoFlo XDP sorter. FACS data were analyzed using FlowJo software.

#### RNA-Seq Analysis

Total RNA was extracted from the cells mentioned above using the Quick-RNA Microprep Kit (Zymo Research, Irvine, CA, USA) according to the manufacturer's protocol. RNA quality was assessed using the Agilent RNA 6000 Nano Kit with the Agilent 2100 Bio analyser (Agilent Technologies, Santa Clara, CA, USA). Library construction and RNA sequencing on the BGISEQ-500 platform were conducted at the Beijing Genomics Institute (BGI). Two biological replicates were performed in RNA-Seq analysis.

The raw sequencing reads were filtered before downstream analyses by removing low-quality reads, adaptor-polluted reads, and reads with more than 10% of unknown bases. Hierarchical indexing for spliced alignment of transcripts (HISAT) was used to map reads to the mm 9 reference genome. Gene expression levels were quantified using RSEM. The gene expression cluster and volcano plot were displayed with R. The DEseq2 algorithms were used to detect differentially expressed genes (DEGs) between groups. Genes with a fold change ≥ 2 and adjusted *P* value ≤ 0.05 were considered to be significantly differentially expressed. Using Gene Ontology (GO) annotation, we classified DEGs according to the official classification and performed GO functional enrichment using gene set enrichment analysis.

## Results

### Transcriptomic Analysis of T Cell Populations in Young and Old Mice

**Figure 1:**
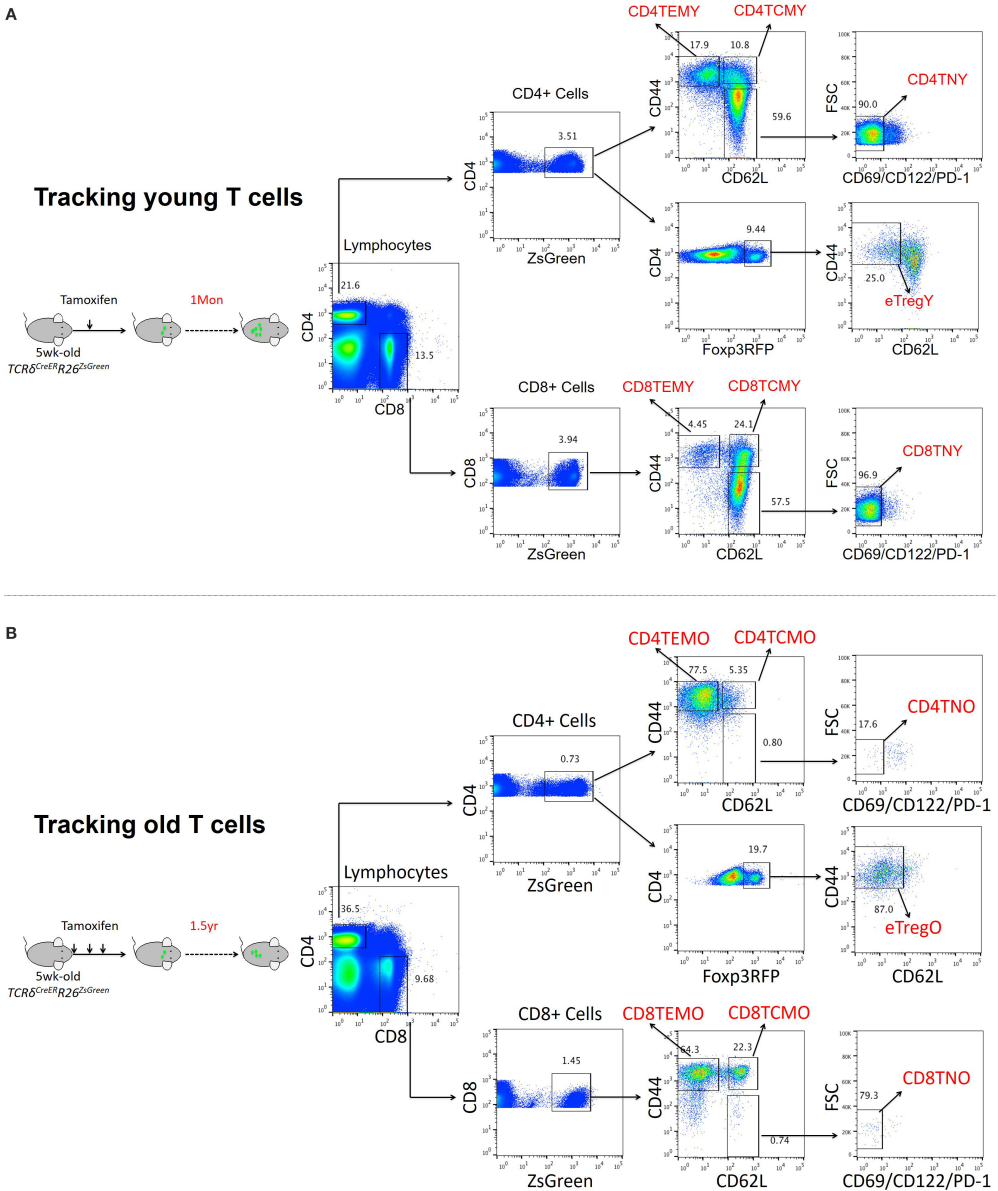
Transcriptomic analysis of T cell populations in young and aged cell populations. **(A,B)** Representative FACS and sorting strategy of ZsGreen-labeled CD4 TEM, CD4 TCM, CD8 TEM, and CD8 TCM cells from *TCR*δ^*CreER*^*R26*^*ZsGreen*^ double transgenic mice treated with tamoxifen for 1 month (young) **(A)** or 1 1/2 years (old) **(B)**.

To fully characterize the effects of aging on T cell phenotype and function, genome-wide transcriptomic analysis was performed to reveal the gene expression profiles of naïve and memory T cells in young and old mice. To obtain T cell populations, *TCR*δ^*CreER*^*R26*^*ZsGreen*^ double transgenic mice developed in our previous work were used (Zhang et al., 2016). For both young and old T cells, *TCR*δ^*CreER*^*R26*^*ZsGreen*^ mice were treated with tamoxifen at 5 weeks old to induce ZsGreen expression. While young T cells were obtained from mice 1 month after treatment, old T cells were obtained from mice 1 1/2 years after treatment (Figures 1A,B). Only small fractions of T cells were ZsGreen positive among both CD4 (3.51%) and CD8 (3.94%) T cells in young mice, and the percentage was even lower in old mice (0.73% for CD4 and 1.45% for CD8 T cells) (Figures 1A,B). Additionally, T cell sub-populations were further identified mainly by CD62L and CD44 expression, in which naïve T cells were CD62L^+^CD44^−^CD69^−^CD122^−^PD-1^−^, central memory T cells (TCM) were CD62^+^CD44^+^, and effector memory T cells (TEM) were CD62^−^CD44^+^. In agreement with the common knowledge that the majority of T cells in old mice are antigen-experienced, naïve T cells and memory T cells were significantly decreased and increased, respectively, in old mice, especially CD44^+^ TEM cells (Figures 1A,B).

Naïve, TCM, and TEM of both CD4^+^ and CD8^+^ T cells were sorted from young and old mice for transcriptome analysis. Compared to young mice, the DEGs in T cells from old mice are summarized in Supplementary Figure 1. For instance, by comparing transcriptomes of young and old CD4 naïve T cells, we found that 6,308 genes were differentially expressed (greater than twofold, *P* < 0.05) with 463 upregulated genes and 5,845 downregulated genes. Among the six T cell populations, significantly more downregulated genes than upregulated ones were observed in old mice in five T cell populations, except for CD8 TEM cells.

With the aging of cells, the number of upregulated genes in CD4 (920, 3.05%) and CD8 (1,873, 5.56%) effector memory T cells (TEM) was significantly much more than that in other subsets. Among downregulated genes, naïve CD4 (5,845) and CD8 (5,494) T cells and CD8 central memory T cells (TCM, 5,523) were remarkably affected by aging, demonstrated by more genes that downregulate significantly. It should be noted that naïve CD4 and CD8 T cells had lower unchanged gene number than other cell subsets, 16,735, 72.63% and 20,857, 78.66%, respectively, which indicated that naïve T cells were sensitive to aging.

### Biological Characteristics of Downregulated Genes in Aged Naïve T Cells

**Figure 2:**
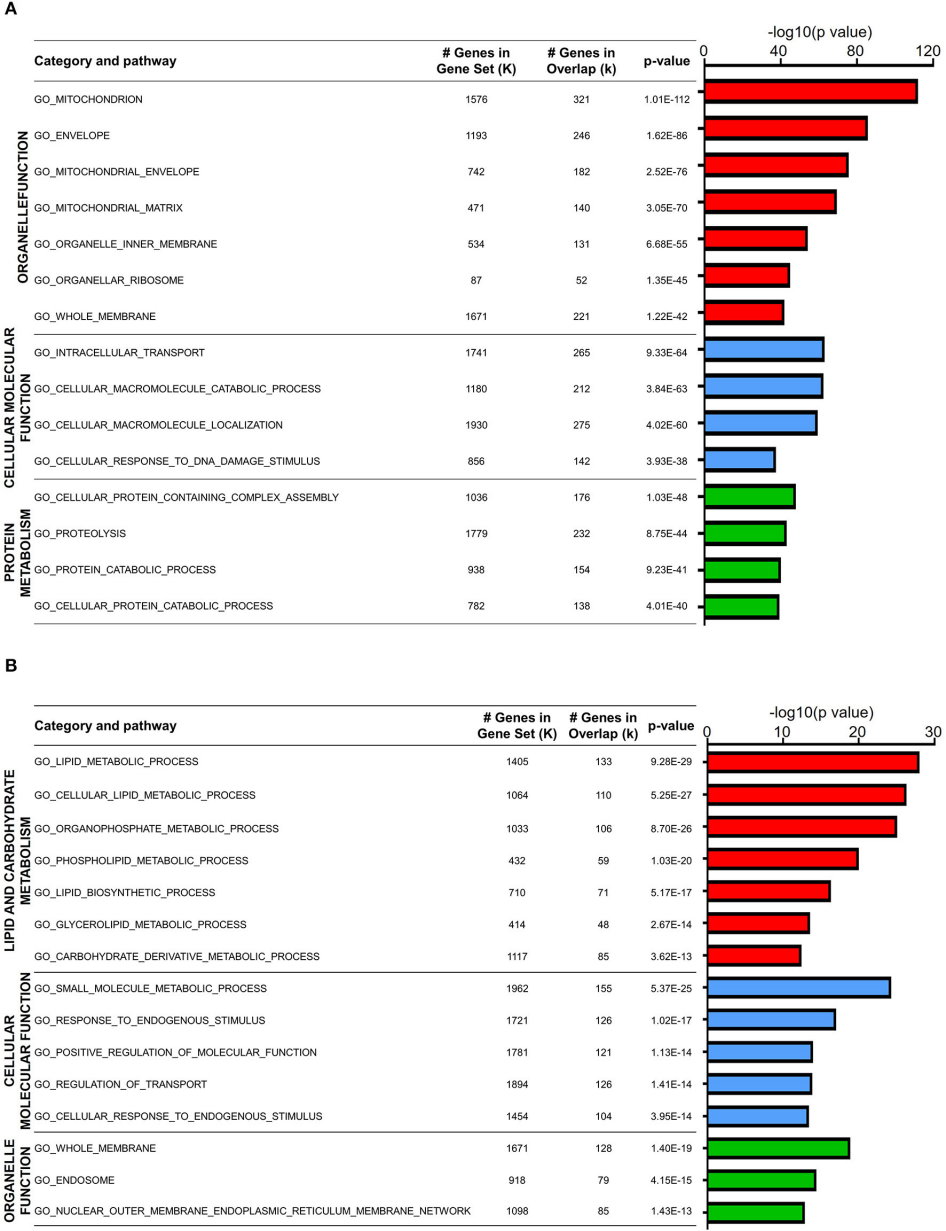
Biological characteristics of downregulated genes in aged naïve T cells. **(A,B)** Gene Ontology analysis of the downregulated DEGs from aged and young naïve CD4 **(A)** and CD8 **(B)** T cells. Column color represents category of pathway. The *X*-axis refers to –log10-transformed *P* value. The table on the right shows GO terms, total gene number, and overlap gene number in the gene set and significance of this term.

Since DEGs in both naïve CD4 and CD8 T cells were mainly downregulated in old mice, we first analyzed the expression features of these genes. GO analysis showed that a large fraction of DEGs in aged naïve CD4 T cells were enriched in three categories: organelle function, cellular molecular function, and protein metabolism (Figure 2A). The total number of genes in each gene set, the overlapped DEGs, and the statistics of *P* value between young and old mice are listed in the figure. Organelle functions are biological processes associated with organelle assembly, such as mitochondrion, envelope, organelle inner membrane, and ribosome. For mitochondrion, 321 DEGs out of 1,576 total genes were identified in this gene set with statistical significance (*P* value = 1.01E−112). Cellular and molecular functions are biological processes mainly associated with intracellular signals and molecular functions, such as cellular molecule transportation, location, and response to DNA damage stimulus. Moreover, protein metabolism, such as proteolysis and protein catabolic processes, was possibly affected in aged CD4 naïve T cells.

GO analysis of DEGs in aged naïve CD8 T cells was also enriched in three categories: lipid and carbohydrate metabolism, cellular and molecular function, and organelle function (Figure 2B). Similar to aged naïve CD4 T cells, the CD8 counterparts showed altered organelle functions, and cellular and molecular functions as well. Furthermore, aging affects lipid and carbohydrate biosynthetic and metabolic processes in aged naïve CD8 T cells. It has been well accepted that T cells differ greatly in their energy needs and metabolic status during immune response, in which lipid metabolism is abruptly transitioned from rest to massive expansion (Howie et al., 2017).

### Functional Characteristics of Age-Related Differential Gene Expression in TCM Population

**Figure 3:**
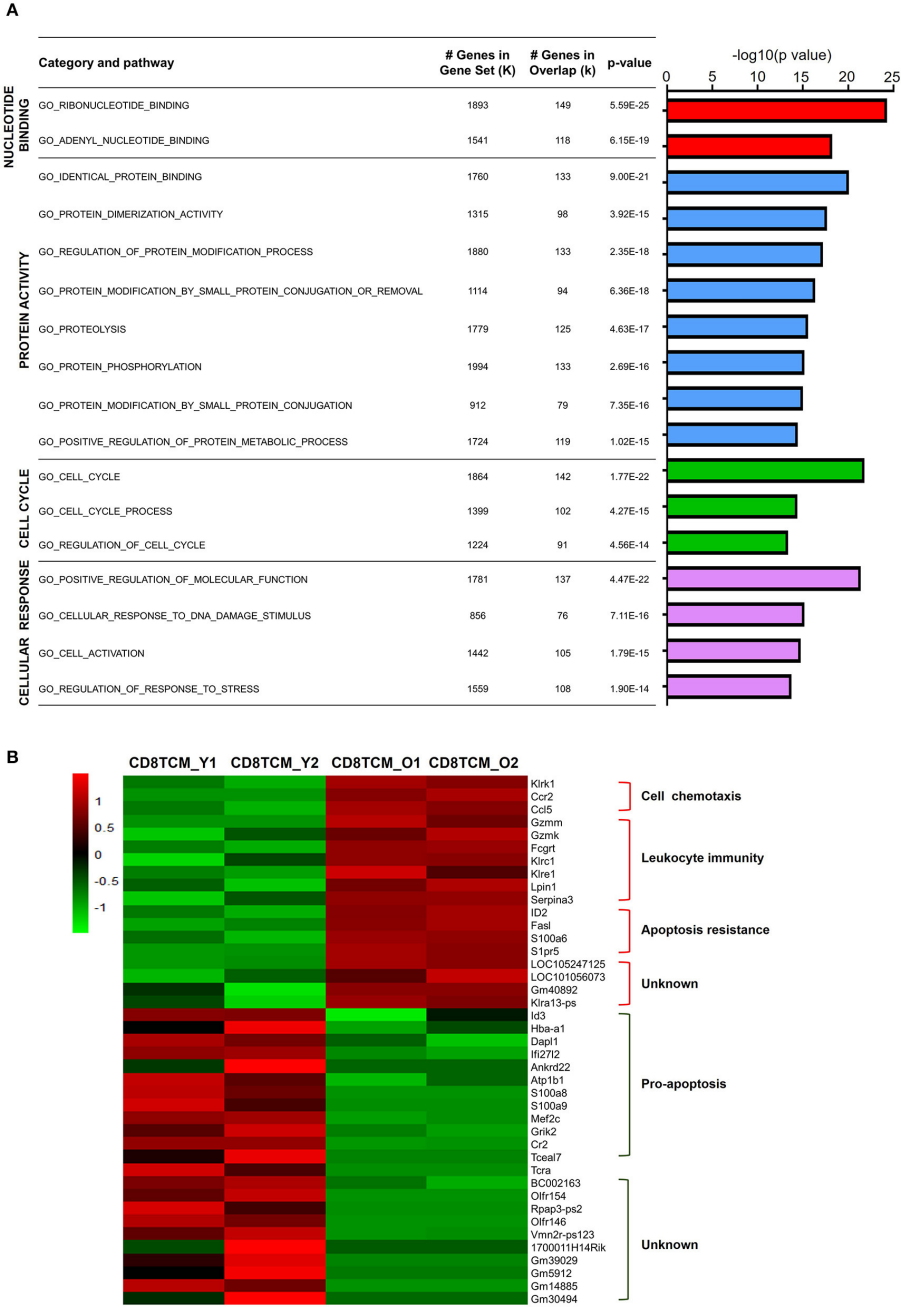
Functional characteristics of age-related differential gene expression in TCM cells. **(A)** Gene Ontology analysis of DEGs from CD4 TCM cells in two groups. The table on the right shows the top four categories. **(B)** Heat map depicts the DEGs of young and aged CD8 TCM cells (*n* = 2). The green and red bands indicate low and high gene expression quantity, respectively.

Characterized by CD62L expression among CD44^+^ antigen-experienced T cells, memory T cells contain distinct populations of CD44^+^CD62L^+^ central memory T cell (TCM) and CD44^+^CD62L^−^ effector memory T cell (TEM) populations (Sallusto et al., 2004). For CD4 TCM cells, 1,641 genes were downregulated, while 426 genes were upregulated. GO analysis of downregulated DEGs in aged CD4 TCM cells revealed that four biological processes were enriched: nucleotide binding, protein activity, cell cycle, and cellular response (Figure 3A). The regulation of DNA binding may be impaired, as evidenced by decreased nucleotide binding genes, such as *Ddx51, Trim23*, and *Rab22a*. Additionally, protein activity-related genes are involved in protein biosynthetic pathways, binding, and dimerization activity, including *Bst2, Snca*, and *Fxr2*. Cell cycle-related genes involved in regulating cell proliferation and apoptosis include *Riok2, Rragb, Ube2e1*, and *Klf4*. It has been reported that *Riok2* promotes cell proliferation (Read et al., 2013) and *Klf4* is a novel tumor suppressor in multiple tumor types (Guan et al., 2010), indicating that cell proliferation was likely to be affected in aged CD4 TCM cells. Furthermore, genes involved in responding to stimulus, including DNA damage and stress, were significantly decreased in aged CD4 TCM cells.

In comparison to aged CD4 TCM cells, the CD8 counterparts showed significantly more changed genes, with 544 upregulated and 5,523 downregulated genes (Supplementary Figure 1). The genes associated with cell chemotaxis, such as chemokine and chemokine receptors CCL5 and CCR2, were elevated (Figure 3B). Interestingly, genes involved in leukocyte immunity were strikingly increased in aged CD8 TCM cells compared to those in young T cells, suggesting an aberrant function in the aged CD8 TCM population. These elevated genes were largely involved in cell cytotoxicity, such as granzyme molecules *Gzmm* and *Gzmk*, Fc receptor *Fcgrt*, and a few killer cell lectin-like receptors *Klrc1, Klre1*, and *Klrk1*. Notably, aging potentially shapes CD8 TCM cell survival and apoptosis signals, endowing them with apoptosis resistance. While genes inhibiting apoptosis were upregulated, such as *Serpina3g, Id2*, and *S1pr5*, a number of pro-apoptotic genes were downregulated in aged CD8 TCM cells, such as *Id3, Dapl1, Ifi27l2, Ankrd22, S100a8, S100a9*, and *Tceal7*.

### Functional Characteristics of Age-Related Gene Expression in TEM Populations

**Figure 4:**
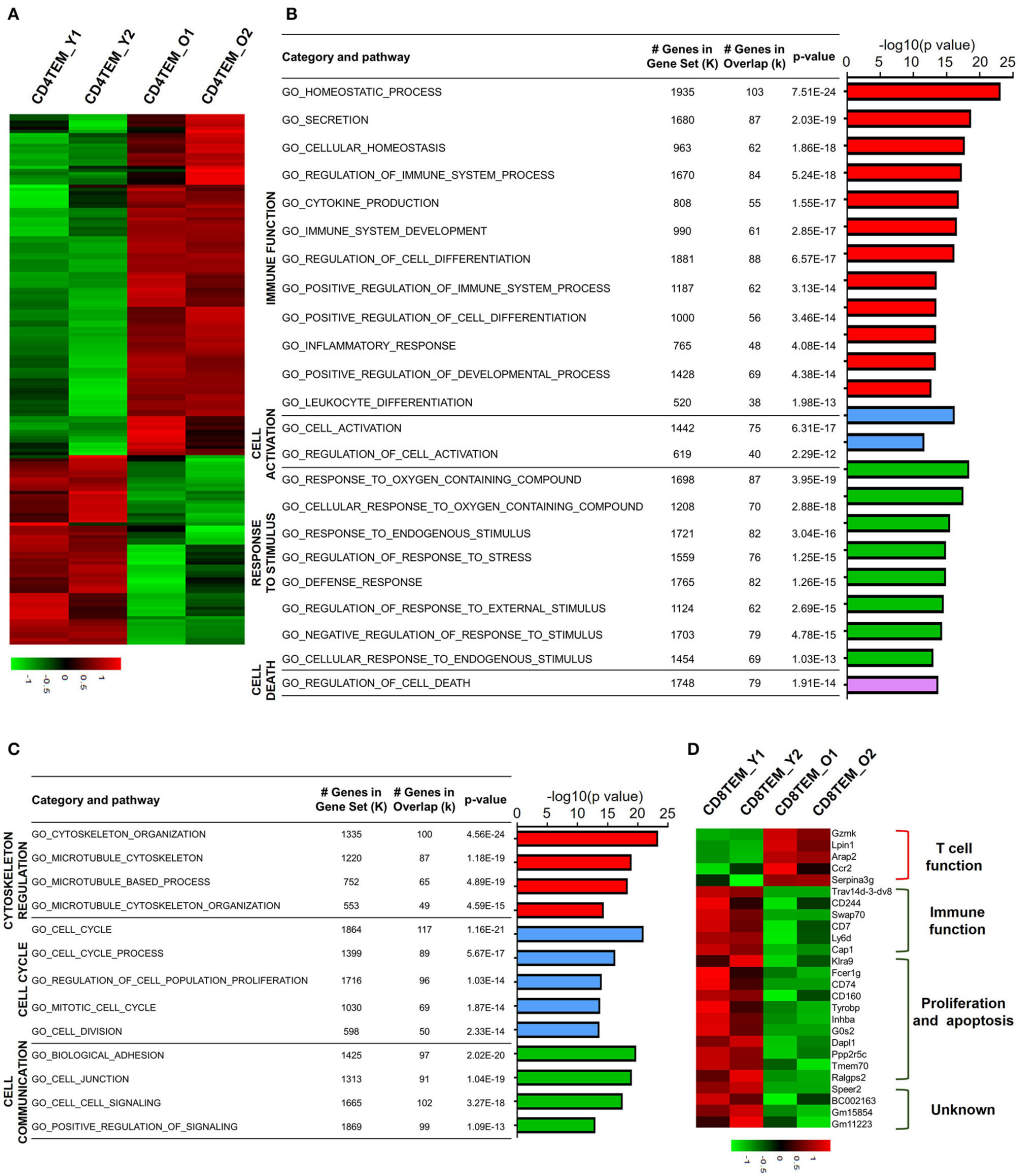
Functional characteristics of age-related differential gene expression in TEM cells. **(A)** Heat map depicts the DEGs of total upregulated and downregulated genes from young and aged CD4 TEM cells. The green and red bands indicate low and high gene expression quantity, respectively. **(B,C)** GO analysis illustrates the activated or suppressed pathways in aged CD4 TEM cells versus young group. **(D)** Heat map depicts a subset of significantly different genes associated with immune function, proliferation, apoptosis, and unknown pathway between young and aged CD8 TEM populations.

Different from TCM cells, effector memory T cells (TEM) are memory T cell populations characterized by rapid effector function, producing cytokines within hours following antigenic stimulation (Sallusto et al., 2004). Aged CD4 TEM cells showed a total 1,542 downregulated genes and 920 upregulated genes compared to young CD4 TEM cells (Figure 4A). GO analysis of upregulated DEGs revealed that mainly four categories of signaling pathways were enriched for immune function, cell activation, response to stimulus, and cell death (Figure 4B). Several GO hits were involved in cell activation, specifically lymphocyte activation. Moreover, signaling pathways associated with T cell immune function regarding lymphocyte differentiation, homeostasis, and cytokine production were also increased in aged CD4 TEM cells, such as *CD274 (PD-L1), CD83, Twsg1, STAT3, RelB, Malt1, Tnfsf8*, and *Tnfrsf4* (*OX40*). Intriguingly, immune checkpoint molecules, molecules that act as gatekeepers of immune responses, were significantly increased, such as *Tigit, CD274* (*PD-L1*), *Pdcd1lg2* (*CD273, PD-L2*), *CTLA-4*, and *Nt5e* (*CD73*). Furthermore, genes involved in response to stimulus were upregulated in aged CD4 TEM cells, such as *Hif1a, Ercc6l2*, and *Ptpn11*. Hif1a is a well-known transcription factor regulated by the presence of oxygen and becomes active under low-oxygen conditions (hypoxia). Hif1a has been intensively studied as a critical regulator of T cell function, disease pathophysiology, and tumor microenvironment, and is thus considered a potential target for drug development (Semenza, 2014). In addition, Ercc excision repair 6 like 2 (*Ercc6l2*) and *Ptpn11* (also known as *Shp2*), which are implicated in reactive oxygen species (ROS) production, were upregulated in aged CD4 TEM cells. These results suggest that aging affects memory T cell functions in response to oxidative stress (Xu et al., 2013; Tummala et al., 2014).

Next, GO analysis revealed that downregulated DEGs in aged CD4 TEM cells were enriched mainly in three categories: cytoskeleton regulation, cell cycle, and cell communication (Figure 4C). The assembly and organization of the cytoskeleton are involved in numerous cellular processes, including T cell activation (Samstag et al., 2003). Cytoskeleton-related genes *Sfrp1, Cdc20, Wdr62, Lrp1*, and *S100a8* were decreased in aged CD4 TEM cells. One of the signs of aging is the arrest of the cell cycle, partially proven by the downregulated expression of a number of cell proliferation-related genes. Survivin, an inhibitor of apoptosis family proteins, not only is thought to be a key molecule in T cell development but also plays a crucial role in T cell proliferation and expansion (Xing et al., 2004). Cenpa is a member of the histone H3 family, and loss of Cenpa leads to a reduction in proliferating phase cells. Importantly, Cenpa is a crucial inhibitor of senescence that can decrease relevant enzyme activity in the heart and cardiac progenitor cells (McGregor et al., 2014). Intriguingly, the expression of *Cenpa* is downregulated in aged CD4 TEM cells, but its role in T cell proliferation, senescence, and apoptosis has not been reported so far.

For aged CD8 TEM cells, only 410 genes were downregulated and 1,873 genes were upregulated, which is in contrast with other subsets. Among the upregulated genes, *Gzmk, Lpin1, Arap2, Ccr2*, and *Serpina3g* were involved in regulating T cell functions. In aged CD8 memory T cells, the granzyme gene *Gzmk* was upregulated, indicating enhanced cytotoxic and killing functions of CD8 T cells (Araki et al., 2009). Serine protease inhibitor 2A (Spi2A), encoded by *Serpina3g*, is a physiological inhibitor of the lysosomal pathway and has been shown to be upregulated in memory CD8 T cells to maintain long-term central memory CD8 T cell populations (Liu et al., 2004). In addition, *Ccr2* (encoding CC chemokine receptor 2) is expressed not only in myeloid cells, such as monocytes, macrophages, and dendritic cells, but also in activated, effector, and memory T cells. Together with its main ligand, chemokine ligand 2 (CCL2), CCR2 controls leukocyte migration during inflammatory processes (Mack et al., 2001). However, the role of upregulation of two other genes, *Lpin1* (encoding magnesium-ion-dependent phosphatidic acid phosphohydrolase enzyme) and *Arap2* (encoding phosphatidylinositol 3,4,5-trisphosphate-dependent GTPase-activating protein), in CD8 memory T cells is unclear.

Analysis of downregulated genes in aged CD8 TEM cells revealed that costimulatory molecules and genes associated with cell proliferation and apoptosis were significantly decreased. CD244 (2B4) and CD160 are usually co-expressed with other inhibitory receptors, including programmed cell death protein-1 (PD-1), cytotoxic T-lymphocyte-associated protein-4 (CTLA-4), lymphocyte-activation gene-3 (Lag-3), and T cell immunoglobulin and mucin domain-3 (Tim-3), as exhausted CD8 T cell markers (Agresta et al., 2018). Expression of CD244 and CD160 was significantly reduced in aged CD8 TEM cells, which possibly resulted in the enhanced abnormal T cell function in these cells (Figure 4D). In addition, the costimulatory molecule CD7 was also downregulated in aged CD8 TEM cells, implying an enhanced effector T cell phenotype with high cytolytic effector molecules and high levels of cytokine production (Aandahl et al., 2003). Similar to CD8 TCM cells, a number of pro-apoptotic genes were downregulated in aged CD8 TEM cells, such as *G0s2, Dapl1, Ppp2r5c*, and *Fcer1g*, indicating that aged CD8 TEM cells tended to be apoptosis resistant.

### Aged Memory T Cells Display Enhanced Leukocyte Immunity Phenotypes

**Figure 5:**
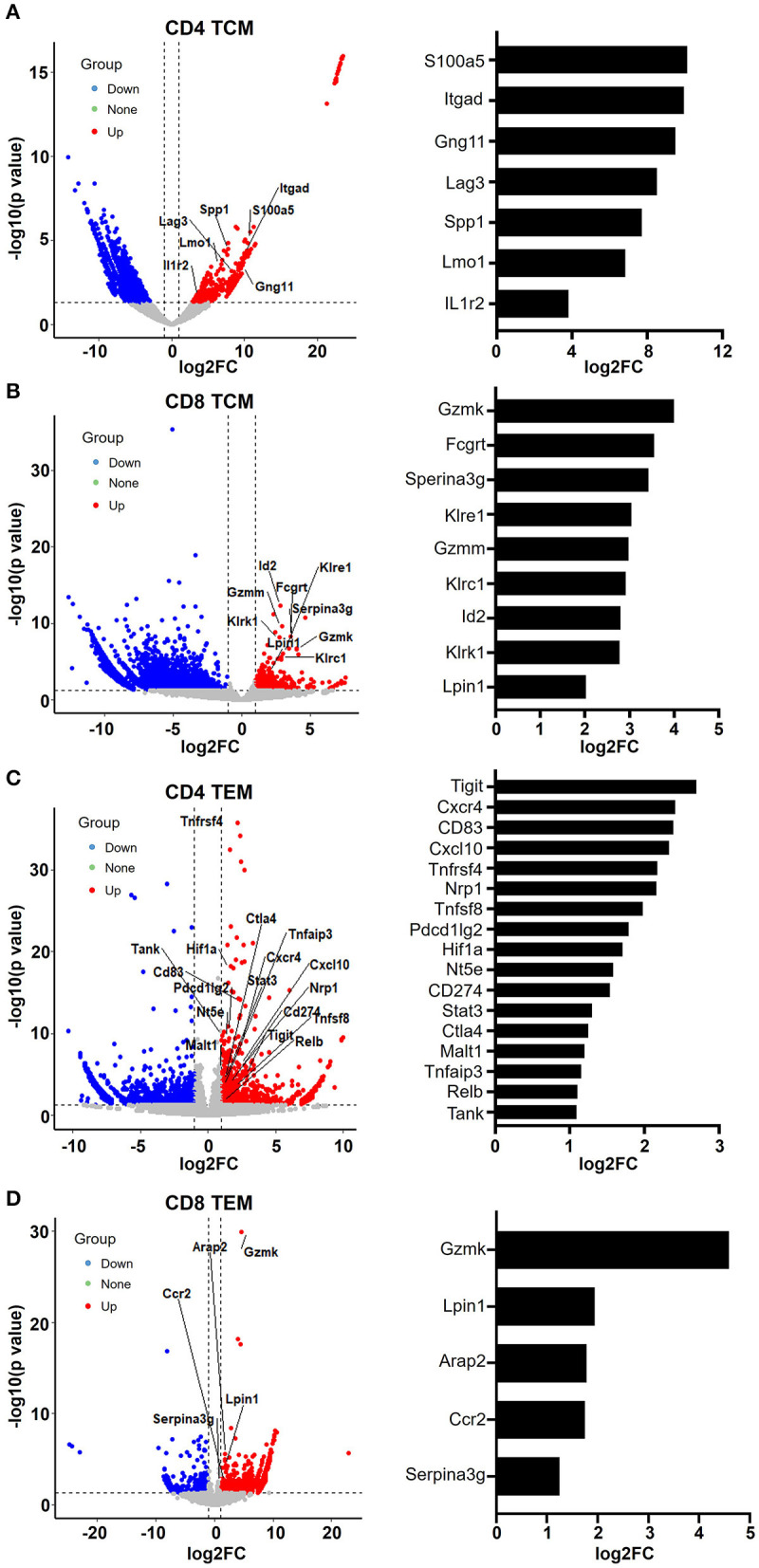
Aging memory T cells display enhanced leukocyte immunity phenotype. **(A–D)** Volcano plots showing differentially expressed genes in CD4 TCM **(A)**, CD8 TCM **(B)**, CD4 TEM **(C)**, and CD8 TEM **(D)** cells from young and aged mice using fold change and *P* value cutoffs of 2 and 0.05, respectively. The labels correspond to upregulated genes involved in leukocyte immunity. The histograms on the right of the volcano plots depict the expression change (log2FC) of these labeled genes.

When we examined the hit signaling pathways and DEGs in aged memory T cells, we observed a remarkable enrichment of genes associated with leukocyte activation, immunity, and exhaustion in both CD4 and CD8 TCM and TEM cells (Figure 5), suggesting that aging causes abnormal T cell functions in memory T cells. In CD4 TCM cells, genes involved in T cell signaling and function were upregulated, such as *Gng11, Itgad, S100a5, Lmo1, Spp1*, and *Csf2ra* (Figure 5A). *Itgad*, encoding the alpha subunit of the cell surface heterodimers, belongs to the beta-2 integrin family and is involved in the activation and adhesion functions of leukocytes (Wu et al., 2004). S100 proteins are calcium-binding proteins localized in the cytoplasm and/or nucleus of a wide range of cells and are involved in the regulation of a number of cellular processes, including inflammation, diseases, and cancer (Bresnick et al., 2015). *Lmo1* encodes a transcriptional regulator, and it has been reported that overexpression of LMO1 inhibits T cell differentiation and induces aggressive T cell acute lymphoblastic leukemia (Greijer and van der Wall, 2004). SPP1 (secreted phosphoprotein 1) acts as a cytokine involved in enhancing the production of interferon gamma and interleukin 12 and reducing the production of interleukin 10, which is essential for the pathway that leads to type I immunity. Meanwhile, aged CD4 TCM cells also showed increased expression of genes related to immunosuppressive T cells, particularly regulatory T cells (Treg), such as *Lag3, Ikzf4, Il1r2*, and *Fgl2*. Lag3 is a well-studied immune checkpoint receptor expressed on Treg cells and plays a critical role in negatively regulating T cell proliferation and activation (Huang et al., 2004). It has been reported that *Ikzf4* and *Il1r2* are preferentially expressed in Treg cells, especially tumor-infiltrating Treg cells (De Simone et al., 2016). *Tigit* in Treg cells induces expression of fibrinogen-like protein 2 (Fgl2), which is an effector molecule, promoting Treg cell-mediated suppression of T effector cell proliferation (Joller et al., 2014).

Similarly, as mentioned earlier, aged CD8 TCM cells showed increased expression of genes important for cytotoxic function of effector CD8 T cells, such as granzyme molecules *Gzmm* and *Gzmk*, Fc receptor *Fcgrt*, and killer cell lectin-like receptors *Klrc1, Klre1*, and *Klrk1* (Figures 3B, 5B). Granzymes are a family of serine proteases essential for inducing cell death in target cells. Killer cell lectin-like receptors (KLRs) are C-type lectin-like receptors that interact specifically with certain HLA class I molecules on cells and are preferentially expressed in cytotoxic killer cells, such as natural killer (NK) and CD8 T cells. According to their function, KLRs are classified as inhibitory and stimulatory receptors. The gene *Klrc1* (encodes NKG2A) is an inhibitory receptor in the KLR family, which is enhanced in tumor-infiltrating CD8 T cells (Chen et al., 2019). In contrast, *Klrk1* (encodes NKG2D) is an activating receptor that promotes CD8 T cell priming, activation, and cytokine production (Kavazovic et al., 2017). However, the role of *Klre1* (encodes NKG2I) is controversial. It was first reported that NKG2I inhibited NK cell cytotoxicity by forming an immunoreceptor tyrosine-based inhibitory motif (ITIM)-bearing partner. Later, it was shown that NKG2I was an activating receptor mediating the recognition and rejection of allogeneic target cells (Koike et al., 2004). This inconsistency may be due to different experimental assays and models. Likewise, the exact functional alterations in aged CD8 TCM cells require comprehensive in-depth investigations.

Analysis of upregulated genes in old CD4 TEM cells revealed that genes associated with T cell activation and immunity were significantly increased, several of which were involved in NF-κB signaling pathways, such as *Tnfsf8, Tnfrsf4, RelB, Malt1, Tank, Tnfaip3, STAT3*, and *Nfatc1* (Figure 5C). Additionally, immune checkpoint molecules, such as *Tigit, Cd274* (*PD-L1*), *Pdcd1lg2* (*CD273, PD-L2*), *Ctla4*, and *Nt5e* (*CD73*), were particularly elevated in aged mice.

In aged CD8 TEM cells, the upregulation of granzyme gene *Gzmk* reflects enhanced cytotoxic and killing functions (Araki et al., 2009). Serine protease inhibitor 2A (Spi2A), encoded by the *Serina3g* gene, is a physiological inhibitor of the lysosomal pathway and has been shown to be upregulated in memory CD8 T cells to maintain long-term central memory populations. In addition, *Ccr2* (encoding CC chemokine receptor 2) not only is expressed in myeloid cells, such as monocytes, macrophages, and dendritic cells, but has also been shown to be expressed in activated, effector, and memory T cells. Together with its main ligand, chemokine ligand 2 (CCL2), CCR2 controls leukocyte migration during inflammatory processes (Mack et al., 2001). However, the roles of upregulation of two other genes, *Lpin1* (encoding magnesium-ion-dependent phosphatidic acid phosphohydrolase enzyme) and *Arap2* (encoding phosphatidylinositol 3,4,5-trisphosphate-dependent GTPase-activating protein), in CD8 memory T cells remain unclear.

### CD4 and CD8 Memory T Cells Exhibit an Inverse Pattern of Apoptotic Gene Expression

Naïve T cells, including both CD4 and CD8 T cells, were significantly decreased in aged mice (Figure 1). GO analysis of DEGs revealed that few genes associated with cell proliferation or apoptosis differed in naïve T cells of young and aged mice (Figure 2). However, a large number of genes related to proliferation and apoptosis were remarkably altered in memory T cells, including both central memory and effector memory CD4 and CD8 T cells in aged mice (Figures 3, 4). Interestingly, while central memory and effector memory T cells showed consistent changes in cell survival and death, CD4 and CD8 memory T cells expressed pro- and anti-apoptotic gene signatures, respectively.

**Figure 6:**
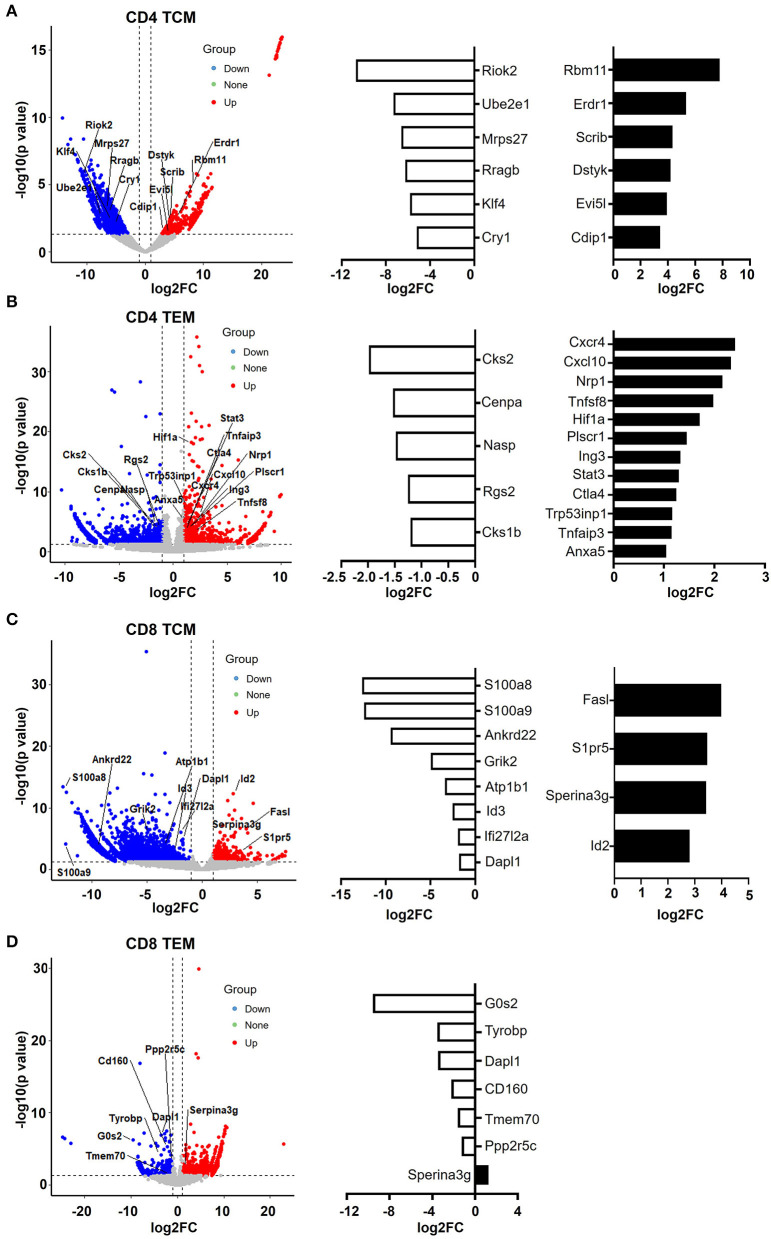
CD4 and CD8 memory T cells have pro- and anti-apoptotic gene patterns, respectively. **(A–D)** Volcano plot showing the gene signature of aged compared to young CD4 TCM **(A)**, CD4 TEM **(B)**, CD8 TCM **(C)**, and CD8 TEM **(D)** cells. *X*-axis represents log2-transformed fold change. *Y*-axis represents –log10-transformed significance. Red points represent upregulated DEGs. Blue points represent downregulated DEGs. Gray points represent non-DEGs. Genes associated with apoptosis are labeled and highlighted.

In CD4 TCM cells, genes that promote cell apoptosis were significantly upregulated, such as *Erdr1, Evi5l, Dstyk, Scrib, Cdip1*, and *Rbm11* (Figure 6A). It has been observed that *Erdr1* induces murine melanoma apoptosis through the regulation of *Bcl-2* and *Bax* (Lee et al., 2016). *Dstyk* induces apoptosis regulated by FGF signaling (Zha et al., 2004). *Scrib*, encoding tumor suppressor Scribble, has been proposed to be important for providing survival advantages in several experimental settings (Frank et al., 2012). Additionally, *Cdip1* is a novel *p53* target gene and is a key downstream effector of *p53*-dependent apoptosis. By interacting with Bap31 at the ER membrane, Cdip transduces the mitochondrial apoptosis pathway (Namba et al., 2013). Accordingly, genes promoting the cell cycle were downregulated (Figure 6A). For example, Riok2 is a member of the RIO (right open reading frame) kinase family and is downregulated in old CD4 TCM cells. Moreover, *Klf4*, characterized as a novel tumor suppressor in multiple tumor types, is significantly decreased, indicating that CD4 TCM cells are susceptible to cell death in aged mice (Guan et al., 2010).

Furthermore, aged CD4 TEM cells also had a large number of pro-apoptotic genes increased, such as *CXCR4, CXCL10, CTLA4, Tnfaip3, STAT3, Tnfsf8, Plscr1, Nrp1, Hif1a, Tp53inp1*, and *Ing3* (Figure 6B). *Tnfsf8* (encoding CD153, also known as CD30L) belongs to the TNF family and is the only known ligand for CD30. CD30L is expressed on the cell surface of activated T cells, and CD30/CD30L interactions induce T cell apoptosis, preventing autoimmunity (Tinazzi et al., 2014). Additionally, Tp53inp1, a TP53-responsive factor, has been shown to positively regulate tumor cell apoptosis (Zuckerberg et al., 1994). Similarly, Ing3 is a tumor suppressor protein that can interact with TP53, inhibit cell growth, and induce apoptosis (Jafarnejad and Li, 2012). Interestingly, AnxA5 (Annexin A5), a calcium- and phospholipid-binding protein, was upregulated in aged CD4 TEM cells. Due to its preferential phosphatidylserine (PS)-binding property, AnxA5 has been utilized as the gold standard for the detection of apoptosis (Krysko et al., 2008; Kang et al., 2020). Recently, AnxA5 expression in CD4 T cells has been shown to be involved in regulatory function and anti-tumor immunotherapy (Kang et al., 2020). Hypoxia-inducible factor (HIF)-1 alpha is the major transcription factor specifically activated by hypoxia. Apoptosis can be induced in response to hypoxia by distinct mechanisms (Greijer and van der Wall, 2004). There is no direct evidence on the role of Hif-1α in CD4 TEM cells; however, it has been shown that hypoxia induces T cell apoptosis by inhibiting lymphocyte expression of IL-2 and CCR7 (Sun et al., 2010). In addition, Hif-1α can also be activated in non-hypoxic conditions, such as during human immunodeficiency virus (HIV)-1 infection in CD4 T cells, in which CD4 T cells are largely depleted *via* cell apoptosis (Duette et al., 2018). Taken together, these data suggest that aged CD4 T memory cells, including both TCM and TEM populations, were pro-apoptotic.

In contrast, senescence shapes apoptosis-resistant gene features in CD8 memory T cells (Figure 6C). In aged CD8 TCM, genes that inhibit cell apoptosis were upregulated, such as *Serpina3g, Id2, FasL*, and *S1pr5*. *Serpina3g* [serine protease inhibitor 2A (Spi2A)], an anti-apoptotic cytosolic cathepsin inhibitor, is induced by both IL-15 and IL-7 and is required for the maintenance of central memory CD8 T cell populations. Overexpressing Spi2A increased the number of memory T lymphocytes (Liu et al., 2004). In addition, inhibitor of DNA binding 2 (*Id2*) was demonstrated to be upregulated in CD8 T cells during infection, and Id2-deficient CD8 T cells were highly susceptible to apoptosis, suggesting that *Id2* is an anti-apoptotic gene in CD8 TCM cells (Cannarile et al., 2006). *S1pr5* (Sphingosine-1-phosphate receptor 5) is the receptor for lysophosphingolipid sphingosine 1-phosphate (S1P), which is proposed to increase cell proliferation and decrease apoptosis in different cells, including memory T cells (He et al., 2019). *FasL*, expressed in memory CD8 T cells and a critical mediator of target cell apoptosis, was increased in aged CD8 TCM cells. Along with the upregulation of anti-apoptotic genes, a number of pro-apoptotic genes were downregulated in aged CD8 TCM cells, such as *Id3, Dapl1, Ifi27l2, Ankrd22, S100a8*, and *S100a9*.

Notably, *Serpina3g* was upregulated in both aged CD8 TCM and CD8 TEM populations (Figures 6C,D), strongly indicating that CD8 memory T cells were anti-apoptotic. In agreement with this, a number of pro-apoptotic genes were downregulated in aged CD8 TEM cells, such as *G0s2, Tyrobp, Dapl1, Tmem70, CD160*, and *Ppp2r5c*. For example, *G0s2* (encodes G0/G1 switch 2) promotes apoptosis by interacting with Bcl2 and preventing the formation of Bcl2/Bax heterodimers (Ohkawara et al., 2013). As its name indicates, *Dapl1* (death-associated protein-like 1) plays a critical role in regulating cell apoptosis. Downregulation of *Dapl1* promotes cell survival and resistance to cell death (Singh et al., 2016). Taken together, aging causes different cell fates for CD4 and CD8 memory T cells, promoting cell apoptosis in CD4 T memory cells while promoting cell survival in CD8 memory T cells.

## Discussion

It is generally accepted that the efficiency of immune functions declines with age, as evidenced by the increased frequency and lethality of infectious diseases, cancer, and autoimmune diseases. In addition, the immune response to vaccination is also impaired with age. Impaired immunity has long been associated with decreased T cell immunity in the elderly (Nikolich-Zugich, 2018). Age-associated decline of T cell function is complex and occurs at multiple levels. To better understand the functional changes in T cells that are associated with senescence, we investigated alterations in gene expression in aged T cells.

The major novelty of the current study is that, other than directly sorting T cell subsets from young and old mice, in which those newly developed T cell populations are contained, we utilized *TCR*δ^*CreER*^*R*26^*ZsGreen*^ double transgenic mice. Tamoxifen treatment induces ZsGreen expression in T cells, making ZsGreen an effective and reliable tracker to trace T cell age. When we sorted out ZsGreen-positive T cell subsets 1 month and 1 1/2 years after treatment, genuine 1-month-old and 1-1/2-year-old T cells were selected, respectively. As shown in Figure 1, only small factions of T cells were ZsGreen positive (<5%) in both young and old mice, demonstrating that T cell turnover is dynamic and most T cell populations are newly developed. Therefore, by utilizing *TCR*δ^*CreER*^*R*26^*ZsGreen*^ mice, we can effectively define and study young and aged T cells.

As senescence affects naïve T cells more profoundly than memory T cells (Haynes et al., 2003), we observed a significant decrease in aged naïve T cells with massive DEGs. GO analysis of downregulated genes in aged T cells revealed that genes related to organelle functions, such as mitochondrion and membrane envelope, were enriched in both naïve CD4 and CD8 T cells. It has been extensively studied that mitochondrial quality and activity are highly associated with aging in a wide range of cells and diseases. In particular, dysfunctional T cell mitochondria lead to inflammatory cytokine production and increased senescence (Desdin-Mico et al., 2020).

The effects of aging on memory T cells were complex and varied in different cell populations between CD4 and CD8 T cells, and between central memory and effector memory T cells. Generally, there were more DEGs in aged CD8 memory T cells than in aged CD4 memory T cells, suggesting that aging largely affects CD8 and central memory T cells. Moreover, the changes in biological processes in aged CD4 TCM cells and TEM cells were inconsistent with respect to cellular response and cell–cell adhesion. All these data suggest that further studies are required to fully elucidate how aging affects T cell adhesion and movement in aged CD4 memory T cells.

Interestingly, we observed a striking enrichment in genes associated with lymphocyte activation and function in all memory T cell populations. For example, in CD4 TCM cells, genes involved in T cell signaling, inflammation, differentiation, and function were upregulated. Similarly, genes associated with T cell activation and immunity were significantly upregulated in aged CD4 TEM, with a large number of genes involved in NF-κB signaling pathways. This is in agreement with a previous report that NF-κB target genes continue to be upregulated in aged CD4 T cells even in the absence of NF-κB induction (Ohkawara et al., 2013). Furthermore, several genes critical for cytotoxic function of effector CD8 T cells, including granzyme molecules and killer cell lectin-like receptors, showed increased expression in both senescent CD8 TCM and TEM cells. Specifically, granzyme gene *Gzmk* was upregulated in aged CD8 memory T cells, indicating enhanced cytotoxic and killing functions of CD8 T memory cells. Notably, we also saw enhanced expression of a cluster of killer cell lectin-like receptors (*Klrc1, Klre1*, and *Klrk1*) associated with NK cell function, which has been shown to be one of the most profound age-related changes in T cells (Chen et al., 2013).

However, enhanced expression of genes associated with T cell function was accompanied by elevated immunosuppressive and exhausted markers, strongly demonstrating aberrant T cell immunity, consistent with a previous study showing that T cell immunity and effector functions in aged mice have defects in response to various pathogens (Nikolich-Zugich et al., 2012). In aged CD4 TCM cells, genes related to immunosuppressive T cells, such as *Lag3* and *Il1r2*, were upregulated. Additionally, a number of immune checkpoint molecules were significantly increased in aged CD4 TEM cells, such as TIGIT, CD274 (PD-L1), PDCD1LG2 (CD273 and PD-L2), CTLA-4, and NT5E (CD73). Upregulation of these inhibitory immune checkpoints exhibited a dominant immunosuppressive function of CD4 memory T cells in old mice, partially explaining the impaired T cell function and reduced vaccine responses and protection in elderly individuals. Knockout or blockade of inhibitory checkpoint pathways could partially restore the functional defect of T cells derived from old mice (Song et al., 2018).

Besides cellular function, aging also affects proliferation and apoptosis processes remarkably and differently in certain memory T cell populations. By analyzing genes associated with cell proliferation and apoptosis in naïve and memory T cell populations of young and old mice, we found that (1) not many altered genes were associated with cell survival and death in naïve T cells; (2) central memory and effector memory T cells had comparable gene changes with respect to cell survival and death within CD4 and CD8 T cells; and (3) CD4 and CD8 memory T cells exhibited pro- and anti-apoptotic gene signatures, respectively. In CD4 TCM cells, genes that promote cell apoptosis, including *Bax, Trp53*, and *mTOR* signaling pathways, were significantly upregulated. There was also a large number of pro-apoptotic genes involved in TNF, p53, and the hypoxia signaling pathway that were increased in aged CD4 TEM cells. The role of Hif-1α in cell apoptosis as an anti-apoptotic or pro-apoptotic factor is controversial, depending on the severity of hypoxia, cell types, and the microenvironment (Piret et al., 2002). Previous studies have demonstrated that Riok2 plays an important role in cell cycle progression, since overexpression of Riok2 promotes cell proliferation, and downregulation of Riok2 causes apoptosis in multiple cell types (Read et al., 2013). The mammalian target of rapamycin (mTOR) is a crucial regulator of cell growth, proliferation, autophagy, and many physiological events, including T cell growth and proliferation (Waickman and Powell, 2012). Ras Related GTP Binding B (RRAGB) plays a critical role in the cellular response through interaction with mTOR protein (Sancak et al., 2008). Taken together, these data suggest that CD4 T memory cells, including both TCM and TEM populations, are prone to cell apoptosis. According to a previous report, the increased cell death in aged memory CD4 T cells may result from increased susceptibility to both Fas- and TNF-mediated apoptosis (Lefebvre and Haynes, 2012). In contrast, it has been shown that senescent CD8 T cells accumulate over time and can constitute more than 50% of the peripheral blood CD8 T cell pool in elderly persons due to their resistance to apoptosis (Effros et al., 2005). In our study, we also noticed gene features with resistance to apoptosis in aged CD8 memory T cells. While genes that inhibit cellular apoptosis were upregulated, those pro-apoptotic genes were downregulated in aged CD8 memory T cells. For example, *Serpina3g* (encoding Spi2A), which is required for the maintenance of long-term memory CD8 T cells, was increased in both old CD8 TCM and CD8 TEM cells, supporting the long-term survival of CD8 T memory cells. The accumulation of senescent CD8 T cells influences the quality of the memory T cell pool, impairs the capacity to respond to vaccination, and is highly associated with age-related disease (Bauer, 2020).

In conclusion, we used *TCR*δ^*CreER*^*R26*^*ZsGreen*^ double transgenic mice and high-throughput mRNA sequencing (RNA-Seq) to study the immunosenescence of naïve and memory T cell populations. A large number of genes involved in cellular and molecular functions, protein activity, cell cycle, cell adhesion, and immune functions were identified as having altered expression during aging. Our work revealed aged CD8 memory T cells with increased T cell activation and immunity genes, yet high expression of immunosuppressive checkpoints and resistance to cell death, implying aberrant T cell immunity in old mice. These feature genes identified in the current study serve as new therapeutic targets for correcting age-related defects.

## Data Availability Statement

The sequencing data has been uploaded to the GEO database (GSE163847, https://www.ncbi.nlm.nih.gov/geo/query/acc.cgi?acc=GSE163847).

## Ethics Statement

The animal study was reviewed and approved by Animal Care Committee of Xi'an Jiaotong University. Written informed consent was obtained from the owners for the participation of their animals in this study.

## Author Contributions

XY, XW (2nd author), LL, LSu, XZho, XZha, CS, and DZ analyzed the data and wrote the original draft. XY, XW (2nd author), KZ, HZ, and JZ performed the FACS analysis. AJ, TX, HL, YS, and CZ collected samples and provided bioinformatic analysis. CS, LSh, XL, XW (17th author), and XZho discussed data analysis.

## Conflict of Interest

The authors declare that the research was conducted in the absence of any commercial or financial relationships that could be construed as a potential conflict of interest.

## Supplementary Material

Supplementary Figure 1 The gene numbers of upregulated, downregulated, and unchanged in aged naïve and memory T cells compared to young T cell population.

## References

[B1] Aandahl E. M. Sandberg J. K. Beckerman K. P. Tasken K. Moretto W. J. Nixon D. F. (2003). CD7 is a differentiation marker that identifies multiple CD8 T cell effector subsets. J. Immunol. 170, 2349–2355. 10.4049/jimmunol.170.5.2349 12594257

[B2] Agresta L. Hoebe K. H. N. Janssen E. M. (2018). The emerging role of CD244 signaling in immune cells of the tumor microenvironment. Front. Immunol. 9:2809. 10.3389/fimmu.2018.02809 30546369 PMC6279924

[B3] Araki Y. Wang Z. Zang C. Wood W. H. III. Schones D. Cui K.. (2009). Genome-wide analysis of histone methylation reveals chromatin state-based regulation of gene transcription and function of memory CD8+ T cells. Immunity 30, 912–925. 10.1016/j.immuni.2009.05.006 19523850 PMC2709841

[B4] Bauer M. E. (2020). Accelerated immunosenescence in rheumatoid arthritis: impact on clinical progression. Immun. Ageing 17:6. 10.1186/s12979-020-00178-w 32190092 PMC7068869

[B5] Bresnick A. R. Weber D. J. Zimmer D. B. (2015). S100 proteins in cancer. Nat. Rev. Cancer 15, 96–109. 10.1038/nrc3893 25614008 PMC4369764

[B6] Cannarile M. A. Lind N. A. Rivera R. Sheridan A. D. Camfield K. A. Wu B. B.. (2006). Transcriptional regulator Id2 mediates CD8+ T cell immunity. Nat. Immunol. 7, 1317–1325. 10.1038/ni1403 17086188

[B7] Chen G. Lustig A. Weng N. P. (2013). T cell aging: a review of the transcriptional changes determined from genome-wide analysis. Front. Immunol. 4:121. 10.3389/fimmu.2013.00121 23730304 PMC3657702

[B8] Chen Y. Xin Z. Huang L. Zhao L. Wang S. Cheng J.. (2019). CD8(+) T cells form the predominant subset of NKG2A(+) cells in human lung cancer. Front. Immunol. 10:3002. 10.3389/fimmu.2019.03002 32010126 PMC6979261

[B9] De Simone M. Arrigoni A. Rossetti G. Gruarin P. Ranzani V. Politano C.. (2016). Transcriptional landscape of human tissue lymphocytes unveils uniqueness of tumor-infiltrating T regulatory cells. Immunity 45, 1135–1147. 10.1016/j.immuni.2016.10.021 27851914 PMC5119953

[B10] Desdin-Mico G. Soto-Heredero G. Aranda J. F. Oller J. Carrasco E. Gabande-Rodriguez E.. (2020). 1371T cells with dysfunctional mitochondria induce multimorbidity and premature senescence. Science 368, 1371–1376. 10.1126/science.aax0860 32439659 PMC7616968

[B11] Dorrington M. G. Bowdish D. M. (2013). Bowdish, immunosenescence and novel vaccination strategies for the elderly. Front. Immunol. 4:171. 10.3389/fimmu.2013.00171 23825474 PMC3695377

[B12] Duette G. Pereyra Gerber P. Rubione J. Perez P. S. Landay A. L. Crowe S. M.. (2018). Induction of HIF-1alpha by HIV-1 infection in CD4(+) T cells promotes viral replication and drives extracellular vesicle-mediated inflammation. mBio 9. 10.1128/mBio.00757-18 30206166 PMC6134101

[B13] Effros R. B. Dagarag M. Spaulding C. Man J. (2005). The role of CD8+ T-cell replicative senescence in human aging. Immunol. Rev. 205, 147–157. 10.1111/j.0105-2896.2005.00259.x 15882351

[B14] Frank S. R. Bell J. H. Frodin M. Hansen S. H. (2012). A betaPIX-PAK2 complex confers protection against Scrib-dependent and cadherin-mediated apoptosis. Curr. Biol. 22, 1747–1754. 10.1016/j.cub.2012.07.011 22863318 PMC3470768

[B15] Greijer A. E. van der Wall E. (2004). The role of hypoxia inducible factor 1 (HIF-1) in hypoxia induced apoptosis. J. Clin. Pathol. 57, 1009–1014. 10.1136/jcp.2003.015032 15452150 PMC1770458

[B16] Guan H. Xie L. Leithauser F. Flossbach L. Moller P. Wirth T.. (2010). Ushmorov, KLF4 is a tumor suppressor in B-cell non-Hodgkin lymphoma and in classic Hodgkin lymphoma. Blood 116, 1469–1478. 10.1182/blood-2009-12-256446 20519630

[B17] Haynes L. Eaton S. M. Burns E. M. Randall T. D. Swain S. L. (2003). CD4 T cell memory derived from young naive cells functions well into old age, but memory generated from aged naive cells functions poorly. Proc. Natl. Acad. Sci. U.S.A. 100, 15053–15058. 10.1073/pnas.2433717100 14657384 PMC299903

[B18] He Y. Shi B. Zhao X. Sui J. (2019). Sphingosine-1-phosphate induces islet beta-cell proliferation and decreases cell apoptosis in high-fat diet/streptozotocin diabetic mice. Exp. Ther. Med. 18, 3415–3424. 10.3892/etm.2019.7999 31602216 PMC6777293

[B19] Howie D. Ten Bokum A. Necula A. S. Cobbold S. P. Waldmann H. (2017). The role of lipid metabolism in T lymphocyte differentiation and survival. Front. Immunol. 8:1949. 10.3389/fimmu.2017.01949 29375572 PMC5770376

[B20] Huang C. T. Workman C. J. Flies D. Pan X. Marson A. L. Zhou G.. (2004). Role of LAG-3 in regulatory T cells. Immunity 21, 503–513. 10.1016/j.immuni.2004.08.010 15485628

[B21] Jafarnejad S. M. Li G. (2012). Regulation of p53 by ING family members in suppression of tumor initiation and progression. Cancer Metastasis Rev. 31, 55–73. 10.1007/s10555-011-9329-5 22095030

[B22] Joller N. Lozano E. Burkett P. R. Patel B. Xiao S. Zhu C.. (2014). Treg cells expressing the coinhibitory molecule TIGIT selectively inhibit proinflammatory Th1 and Th17 cell responses. Immunity 40, 569–581. 10.1016/j.immuni.2014.02.012 24745333 PMC4070748

[B23] Kang T. H. Park J. H. Yang A. Park H. J. Lee S. E. Kim Y. S.. (2020). Annexin A5 as an immune checkpoint inhibitor and tumor-homing molecule for cancer treatment. Nat. Commun. 11:1137. 10.1038/s41467-020-14821-z 32111835 PMC7048819

[B24] Kavazovic I. Lenartic M. Jelencic V. Jurkovic S. N Lemmermann A. W.. (2017). NKG2D stimulation of CD8(+) T cells during priming promotes their capacity to produce cytokines in response to viral infection in mice. Eur. J. Immunol. 47, 1123–1135. 10.1002/eji.201646805 28378389

[B25] Koike J. Wakao H. Ishizuka Y. Sato T. A. Hamaoki M. Seino K.. (2004). Bone marrow allograft rejection mediated by a novel murine NK receptor, NKG2I. J. Exp. Med. 199, 137–144. 10.1084/jem.20030851 14707119 PMC1887729

[B26] Krysko D. V. Vanden Berghe T. D'Herde K. Vandenabeele P. (2008). Apoptosis and necrosis: detection, discrimination and phagocytosis. Methods 44, 205–221. 10.1016/j.ymeth.2007.12.001 18314051

[B27] Lee J. Jung M. K. Park H. J. Kim K. E. Cho D. (2016). Erdr1 suppresses murine melanoma growth *via* regulation of apoptosis. Int. J. Mol. Sci. 17:107. 10.3390/ijms17010107 26784177 PMC4730348

[B28] Lefebvre J. S. Haynes L. (2012). Aging of the CD4 T cell compartment. Open Longev. Sci. 6, 83–91. 10.2174/1876326X01206010083 24839469 PMC4020238

[B29] Li M. Yao D. Zeng X. Kasakovski D. Zhang Y. Chen S.. (2019). Age related human T cell subset evolution and senescence. Immun. Ageing 16:24. 10.1186/s12979-019-0165-8 31528179 PMC6739976

[B30] Liu N. Phillips T. Zhang M. Wang Y. Opferman J. T. Shah R.. (2004). Serine protease inhibitor 2A is a protective factor for memory T cell development. Nat. Immunol. 5, 919–926. 10.1038/ni1107 15311278

[B31] Mack M. Cihak J. Simonis C. Luckow B. Proudfoot A. E. Plachy J.. (2001). Expression and characterization of the chemokine receptors CCR2 and CCR5 in mice. J. Immunol. 166, 4697–4704. 10.4049/jimmunol.166.7.4697 11254730

[B32] McGregor M. Hariharan N. Joyo A. Y. Margolis R. L. Sussman M. A. (2014). CENP-A is essential for cardiac progenitor cell proliferation. Cell Cycle 13, 739–748. 10.4161/cc.27549 24362315 PMC3979910

[B33] Namba T. Tian F. Chu K. Hwang S. Y. Yoon K. W. Byun S.. (2013). CDIP1-BAP31 complex transduces apoptotic signals from endoplasmic reticulum to mitochondria under endoplasmic reticulum stress. Cell Rep. 5, 331–339. 10.1016/j.celrep.2013.09.020 24139803 PMC3833439

[B34] Nikolich-Zugich J. (2018). The twilight of immunity: emerging concepts in aging of the immune system. Nat. Immunol. 19, 10–19. 10.1038/s41590-017-0006-x 29242543

[B35] Nikolich-Zugich J. Li G. Uhrlaub J. L. Renkema K. R. Smithey M. J. (2012). Age-related changes in CD8 T cell homeostasis and immunity to infection. Semin. Immunol. 24, 356–364. 10.1016/j.smim.2012.04.009 22554418 PMC3480557

[B36] Ohkawara T. Oyabu A. Ida-Eto M. Tashiro Y. Narita N. Narita M. (2013). Subtype-specific parafollicular localization of the neuropeptide manserin in the rat thyroid gland. Acta Histochem. 115, 190–194. 10.1016/j.acthis.2012.05.003 22682498

[B37] Piret J.-P. Mottet D. Raes M. Michiels C. (2002). Is HIF-1α a pro- or an anti-apoptotic protein? Biochem. Pharmacol. 64, 889–892. 10.1016/S0006-2952(02)01155-3 12213583

[B38] Read R. D. Fenton T. R. Gomez G. G. Wykosky J. Vandenberg S. R. Babic I.. (2013). A kinome-wide RNAi screen in *Drosophila glia* reveals that the RIO kinases mediate cell proliferation and survival through TORC2-Akt signaling in glioblastoma. PLoS Genet. 9:e1003253. 10.1371/journal.pgen.1003253 23459592 PMC3573097

[B39] Sallusto F. Geginat J. Lanzavecchia A. (2004). Central memory and effector memory T cell subsets: function, generation, and maintenance. Annu. Rev. Immunol. 22, 745–763. 10.1146/annurev.immunol.22.012703.104702 15032595

[B40] Samstag Y. Eibert S. M. Klemke M. Wabnitz G. H. (2003). Actin cytoskeletal dynamics in T lymphocyte activation and migration. J. Leukoc. Biol. 73, 30–48. 10.1189/jlb.0602272 12525560

[B41] Sancak Y. Peterson T. R. Shaul Y. D. Lindquist R. A. Thoreen C. C. Bar-Peled L. (2008). The Rag GTPases bind raptor and mediate amino acid signaling to mTORC1. Science 320, 1496–1501. 10.1126/science.1157535 18497260 PMC2475333

[B42] Saule P. Trauet J. Dutriez V. Lekeux V. Dessaint J. P. Labalette M. (2006). Accumulation of memory T cells from childhood to old age: central and effector memory cells in CD4(+) versus effector memory and terminally differentiated memory cells in CD8(+) compartment. Mech. Ageing Dev. 127, 274–281. 10.1016/j.mad.2005.11.001 16352331

[B43] Semenza G. L. (2014). Oxygen sensing, hypoxia-inducible factors, and disease pathophysiology. Annu. Rev. Pathol. 9, 47–71. 10.1146/annurev-pathol-012513-104720 23937437

[B44] Singh P. Ravanan P. Talwar P. (2016). Death associated protein kinase 1 (DAPK1): a regulator of apoptosis and autophagy. Front. Mol. Neurosci. 9:46. 10.3389/fnmol.2016.00046 27445685 PMC4917528

[B45] Song Y. Wang B. Song R. Hao Y. Wang D. Li Y.. (2018). T-cell immunoglobulin and ITIM domain contributes to CD8(+) T-cell immunosenescence. Aging Cell. 17:e12716. 10.1111/acel.12716 29349889 PMC5847879

[B46] Sun J. Zhang Y. Yang M. Zhang Y. Xie Q. Li Z.. (2010). Hypoxia induces T-cell apoptosis by inhibiting chemokine C receptor 7 expression: the role of adenosine receptor A(2). Cell Mol. Immunol. 7, 77–82. 10.1038/cmi.2009.105 20029460 PMC4003256

[B47] Thome J. J. Grinshpun B. Kumar B. V. Kubota M. Ohmura Y. Lerner H.. (2016). Longterm maintenance of human naive T cells through in situ homeostasis in lymphoid tissue sites. Sci. Immunol. 1:aah6506. 10.1126/sciimmunol.aah6506 28361127 PMC5367636

[B48] Tinazzi E. Barbieri A. Rigo A. Patuzzo G. Beri R. Gerli R.. (2014). In rheumatoid arthritis soluble CD30 ligand is present at high levels and induces apoptosis of CD30(+)T cells. Immunol. Lett. 161, 236–240. 10.1016/j.imlet.2014.01.007 24447865

[B49] Tummala H. Kirwan M. Walne A. J. Hossain U. Jackson N. Pondarre C.. (2014). ERCC6L2 mutations link a distinct bone-marrow-failure syndrome to DNA repair and mitochondrial function. Am. J. Hum. Genet. 94, 246–256. 10.1016/j.ajhg.2014.01.007 24507776 PMC3928664

[B50] Vermes I. Haanen C. Steffensnakken H. Reutelingsperger C. A. (1995). Novel assay for apoptosis—flow cytometric detection of phosphatidylserine expression on early apoptotic cells using fluorescein-labeled Annexin-V. J. Immunol. Methods 184, 39–51. 10.1016/0022-1759(95)00072-I 7622868

[B51] Waickman A. T. Powell J. D. (2012). mTOR, metabolism, and the regulation of T-cell differentiation and function. Immunol. Rev. 249, 43–58. 10.1111/j.1600-065X.2012.01152.x 22889214 PMC3419491

[B52] Wu H. Rodgers J. R. Perrard X. Y. Perrard J. L. Prince J. E. Abe Y.. (2004). Deficiency of CD11b or CD11d results in reduced staphylococcal enterotoxin-induced T cell response and T cell phenotypic changes. J. Immunol. 173, 297–306. 10.4049/jimmunol.173.1.297 15210787

[B53] Xing Z. Conway E. M. Kang C. Winoto A. (2004). Essential role of survivin, an inhibitor of apoptosis protein, in T cell development, maturation, and homeostasis. J. Exp. Med. 199, 69–80. 10.1084/jem.20031588 14699085 PMC1887718

[B54] Xu D. Zheng H. Yu W. M. Qu C. K. (2013). Activating mutations in protein tyrosine phosphatase Ptpn11 (Shp2) enhance reactive oxygen species production that contributes to myeloproliferative disorder. PLoS ONE 8:e63152. 10.1371/journal.pone.0063152 23675459 PMC3651249

[B55] Zha J. Zhou Q. Xu L. G. Chen D. Li L. Zhai Z.. (2004). RIP5 is a RIP-homologous inducer of cell death. Biochem. Biophys. Res. Commun. 319, 298–303. 10.1016/j.bbrc.2004.04.194 15178406

[B56] Zhang B. Jia Q. Bock C. Chen G. Yu H. Ni Q.. (2016). Zhuang, glimpse of natural selection of long-lived T-cell clones in healthy life. Proc. Natl. Acad. Sci. U.S.A. 113, 9858–9863. 10.1073/pnas.1601634113 27535935 PMC5024599

[B57] Zuckerberg A. L. Goldberg L. I. Lederman H. M. (1994). Effects of hypoxia on interleukin-2 mRNA expression by T lymphocytes. Crit. Care Med. 22, 197–203. 10.1097/00003246-199402000-00008 8306676

