# Supplementary figures and images for: Age-Related Gene Alteration in Naïve and Memory T cells Using Precise Age-Tracking Model
Source: Front Cell Dev Biol. 2021 Feb 11;8:624380. doi: 10.3389/fcell.2020.624380 (PMC7905051; doi:10.3389/fcell.2020.624380)

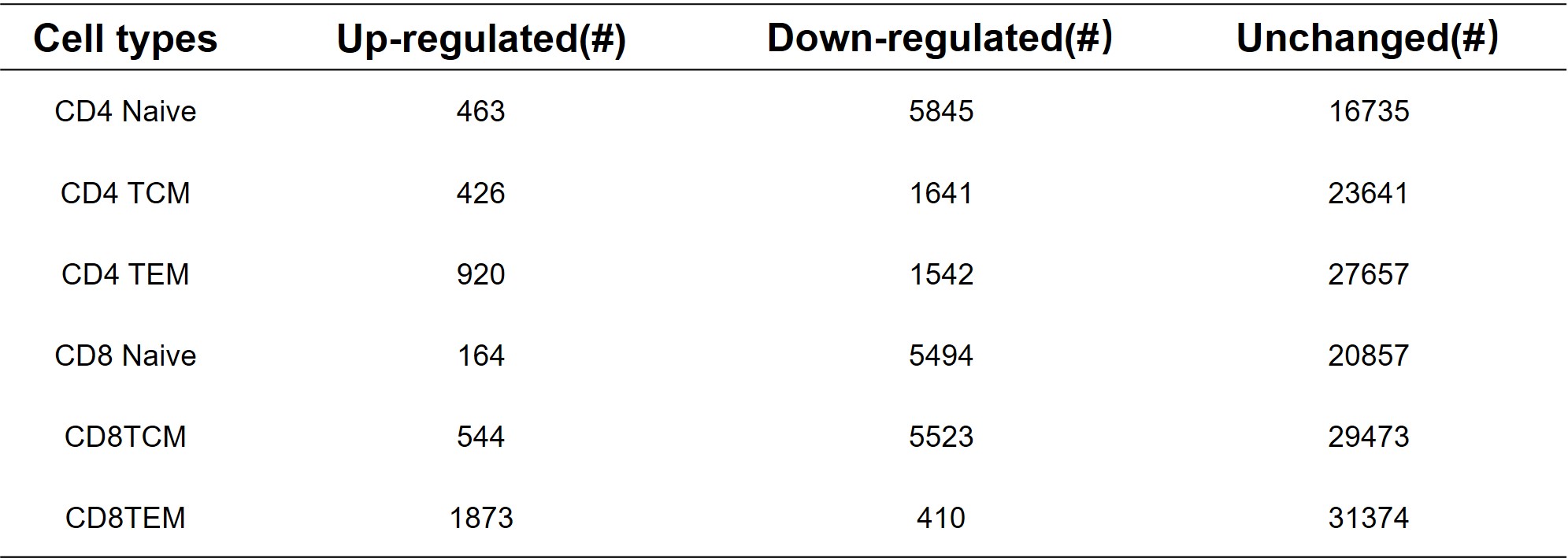

Supplement: Supplementary Figure 1 — The gene numbers of upregulated, downregulated, and unchanged in aged naïve and memory T cells compared to young T cell population. [file Image_1.JPEG]
